# HBc: the multifunctional architect of HBV replication, immune evasion, and therapeutic innovation

**DOI:** 10.3389/fimmu.2025.1657920

**Published:** 2025-11-17

**Authors:** Yujia Zhu, Hongxiao Song, Fengchao Xu, Mian Huang, Guangyun Tan

**Affiliations:** 1 Department of Hepatology, Center of Infectious Diseases and Pathogen Biology, Institute of Translational Medicine, The First Hospital of Jilin University, Changchun, Jilin, China; 2 Jilin Provincial Key Laboratory of Metabolic Liver Diseases, Jilin University, Changchun, Jilin, China; 3 China-Singapore Belt and Road Joint Laboratory on Liver Disease Research, Changchun, Jilin, China

**Keywords:** hepatitis B core protein (HBc), HBV replication, immune evasion, capsidassembly modulators (CAMs), therapeutic target

## Abstract

The hepatitis B core protein (HBc) is a multifunctional viral protein central to hepatitis B virus (HBV) replication, immune modulation, and capsid assembly. Structurally, HBc consists of an N-terminal domain (NTD) essential for capsid formation and a C-terminal domain (CTD) critical for RNA binding and genome packaging. Frequent HBc mutations, driven by HBV’s high mutation rate, enhance the virus’s ability to adapt to environmental pressures. HBc interacts with host factors to regulate viral transcription, stabilize capsids, and modulate immune responses, including the suppression of interferon signaling and promotion of immune exhaustion. Clinically, anti-HBc antibodies serve as key diagnostic markers, while HBc-targeting therapies, such as capsid assembly modulators (CAMs), represent promising strategies for achieving functional cure. This review uniquely integrates structural, functional, and clinical perspectives on HBc, providing a comprehensive understanding of its role in HBV biology and its potential as a therapeutic target. By highlighting recent advances in CAMs and the challenges of drug resistance, this work offers valuable insights for researchers and clinicians aiming to develop innovative HBV treatments.

## 1 Introduction

Chronic hepatitis B virus (HBV) infection remains a major global public health challenge. In 2019, an estimated 296 million individuals worldwide tested positive for hepatitis B surface antigen (HBsAg), with a global prevalence of chronic HBV infection of approximately 3.5%. High-endemic areas include China, Southeast Asia, and sub-Saharan Africa (1). HBV is a hepatotropic virus with a narrow host range, infecting only humans and a few non-human primates. HBV infection can lead to a spectrum of liver diseases, including acute and chronic hepatitis, liver cirrhosis, and hepatocellular carcinoma (HCC), and it is recognized as a leading cause of HCC worldwide (2–4).

There are three types of HBV particles present in the blood of infected individuals: 22 nm diameter spherical and filamentous subviral particles (SVPs) and the 44 nm diameter Dane particles. SVPs are non-infectious but highly immunogenic, whereas Dane particles are infectious (5). Dane particles consist of an envelope made of lipid bilayers embedded with HBsAg and a nucleocapsid. The nucleocapsid is a capsid composed of 180 or 240 hepatitis B core proteins (HBc), which encloses the viral DNA genome and the viral polymerase (Pol) (6). The HBV genome is a 3.2 kb partially double-stranded, relaxed circular DNA (rcDNA), containing a complete negative strand and an incomplete positive strand.

Upon infection, HBV initially binds with low affinity to heparan sulfate proteoglycans (HSPGs) on the host cell surface, facilitating viral concentration on the target cell and increasing the likelihood of receptor engagement (7, 8). High-affinity binding then occurs between the PreS1 domain of the HBV L envelope protein and the sodium taurocholate co-transporting polypeptide (NTCP), leading to NTCP oligomerization and promoting viral internalization and release of the nucleocapsid into the cytoplasm. Neuropilin-1 (NRP1) further enhances viral attachment and facilitates the interaction between PreS1 and NTCP, thus promoting HBV infection (9–11). Subsequently, the rcDNA is transported into the nucleus, where it is converted into covalently closed circular DNA (cccDNA) with the assistance of host DNA repair machinery (12, 13).

The HBV genome contains four overlapping open reading frames (ORFs)—S, C, P, and X—which, after infection and cccDNA formation, serve as transcriptional templates for six partially overlapping viral mRNAs: pregenomic RNA (pgRNA, 3.5 kb), preC RNA (3.5 kb), preS1 HBs RNA (2.4 kb), preS2/S HBs RNA (2.1 kb), and HBV X RNA (0.7 kb) (14). These transcripts encode various viral proteins: pgRNA encodes HBc and Pol; preC RNA encodes hepatitis B e antigen (HBeAg); preS1, preS2 and S HBs RNA encodes the L envelope protein; preS2 and S HBs RNA encodes the M envelope proteins, S HBs RNA encodes the S envelope proteins; and HBV X RNA encodes HBx protein.

HBc forms dimers that assemble into capsids, encapsidating Pol along with pgRNA. Inside the capsid, Pol catalyzes reverse transcription of pgRNA to synthesize rcDNA. Mature nucleocapsids can either be transported back into the nucleus to replenish the cccDNA pool or be enveloped by viral surface proteins within multivesicular bodies (MVBs) and subsequently released from hepatocytes as fully formed infectious virions (15–17).

## 2 Hepatitis B core protein

### 2.1 HBc protein structure

**Figure 1:**
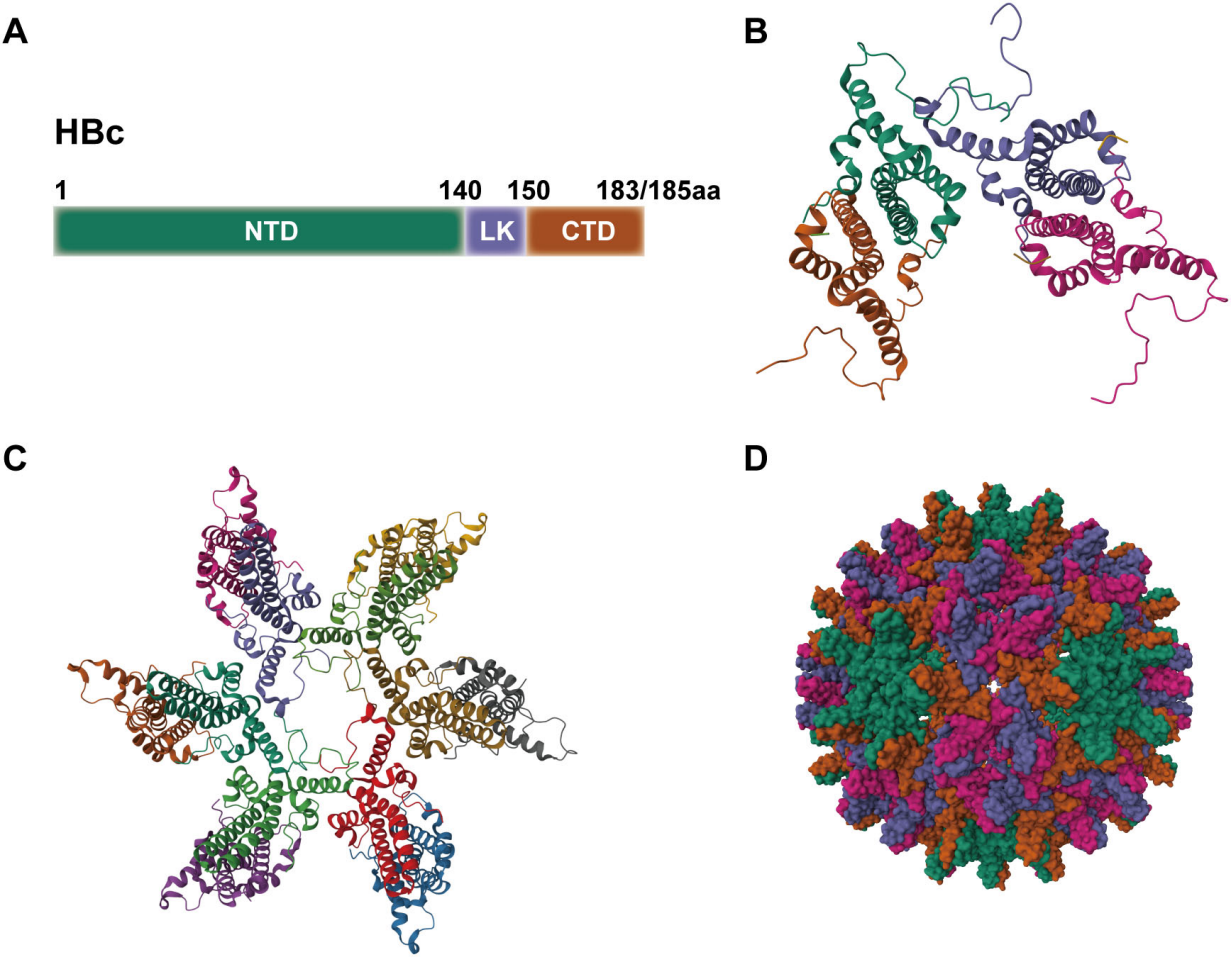
The structure of HBc. **(A)** Domains of the HBc protein. **(B)** Ribbon model of HBc protein. **(C)** Ribbon model of HBV capsid. **(D)** Spherical structure of HBV capsid.

HBc protein consists of 183 or 185 amino acid residues (aa) and contains two structural domains: the N-terminal domain (NTD, aa 1-140) and the C-terminal domain (CTD, aa 150-183/185), which are connected by a linker peptide (aa 141-149) (18). HBc is primarily composed of α-helical structures, with five α-helices. The main feature is a long α-helix formed by residues 50–73 (α3) and 79–110 (α4), which forms a helical hairpin that dimerizes through α-helical interactions (19). HBc exhibits a flexible structure, and the conformation of free HBc dimers differs from the conformation of dimers that form the capsid. It is thus proposed that HBc exists in capsid assembly-active (HBcAct), capsid assembly-incompetent (HBcInc), or abnormal (HBcAbb) conformations, with only HBcAct capable of assembling into icosahedral capsids. In contrast, HBcInc/HBcAbb assemble into non-icosahedral forms, disrupting the HBV life cycle (20, 21). The homodimer of HBc contains two structural domains: the α3 and α4 helices of the monomer, which wrap around each other to form a four-helix bundle dimerization interface linked by disulfide bonds, and the α1, α2, and α5 helices, which surround the base of the four-helix bundle to form the hydrophobic core of the contact domain (21, 22) (**Figure 1**). HBc dimers serve as the building blocks of capsid assembly, which consists of a double-layer structure, with the NTD forming the outer layer and the CTD and its associated RNA forming the inner layer (23).

### 2.2 HBc variants

HBV replicates through reverse transcription of its pgRNA. Due to the lack of proofreading activity in its reverse transcriptase, HBV has a significantly higher mutation rate than other DNA viruses (24). Studies have identified 54 missense mutation sites in the HBc protein in hepatocellular carcinoma tissues, mainly concentrated in three major (codons 83–87, 95–104, and 130–135) and three minor (codons 21–38, 59–63, and 151–155) mutation cluster regions (MCR), with the most frequent mutations being P130T, I97L, and S87G (25).

HBV can evolve through mutations, increasing its adaptability to environmental selection pressures. These mutations may influence various aspects of viral replication and pathogenesis. In the presence of HLA class I alleles, sequence analysis of HBc revealed nine mutated amino acid residues, seven of which are within the CD8+ T cell-targeted epitopes (26). For instance, the L60V mutation in HBc, where leucine is substituted with valine, is a typical HLA-A2-binding peptide, and its affinity for HLA-A*0201 is enhanced. The HLA molecule captures and presents this antigenic peptide to T cells, leading to T cell activation, proliferation, differentiation, and cytotoxic T lymphocyte (CTL) responses that contribute to liver damage. Furthermore, the HBc L60V mutation promotes capsid assembly and enhances HBV replication, but it results in reduced viral secretion. Additionally, a proline deletion at position 5 of HBc also causes low-level viral secretion (27, 28).

The most common natural mutation of HBc occurs at amino acid position 97, where isoleucine or phenylalanine is substituted with leucine. The surface spikes of the capsid contain a hydrophobic pocket formed largely by residues P5, L60, L95, K96, and I97/F97 (29, 30). Bioinformatics and cryo-EM studies revealed that the hydrophobic pocket surrounding the I97L mutation exhibits defects in maintaining tight coupling between genome maturation and viral secretion. The signal for genome maturation is transmitted from the interior of the capsid to its surface via this hydrophobic pocket, and thus the I97L mutant results in excessive secretion of virions containing immature genomes (31). Additionally, the F97L mutation enhances capsid assembly capability (32). Although the P130T mutation does not directly affect the pocket, it increases the maturation of the intracellular genome. Studies have shown that the immature secretion of the I97L mutant can be compensated by the P130T mutation, and the P130T mutation is often associated with the occurrence of I97L mutations (33) (**Table 1**).

**Table 1:** HBc variants and clinical significance.

Variants	Clinical significance	References
P5T	Low-level of viral secretion Resistant to NAs	(27, 144)
L60V	Low-level of viral secretion Enhance viral replication Eliciting a host immune response may lead to liver damage	(27, 28)
I97L	Secretion of too much virus containing an immature genome Inhibits the persistence of the viral genome	(31)
T109I/M/S	Affects the assembly of the capsid Associated with resistance to CAM therapy	(162, 163)
P130L	Increase genome maturation Extend the persistence of the viral genome May be associated with immune evasion	(33, 145)
D29G	Inhibits the antiviral activity of CAM	(164)
T33N/S		
Y118F		
S106T	Enhance the antiviral activity of CAM	(164)
T128I		
L140I		
S35T	Facilitate pgRNA packaging Enhance viral replication May be associated with resistance to NAs	(144)
P79Q		
E83D		
S87G		
Q177K		

## 3 The role of core in viral particle formation

### 3.1 NTD-mediated capsid assembly

The NTD is essential for capsid formation, consisting of five α-helices. Among them, α1, α2, and α5 form a hydrophobic core that stabilizes the monomer structure, while the α3 and α4 helices of two monomers interact through hydrophobic forces to drive the formation of a four-helix bundle, resulting in core dimerization and the formation of a homodimer. This dimer then assembles into a capsid through hydrophobic interactions between dimer-dimer interfaces (34–36). The core dimer can form two types of icosahedral symmetry shells, namely T = 3 or T = 4. The T = 3 capsid, with a diameter of 30 nm, consists of 90 core dimers, while the T = 4 particle, with a diameter of 34 nm, consists of 120 core dimers (15). Studies have shown that the NTD alone is sufficient to assemble a capsid under high HBc and/or salt concentrations, but the stability of the capsid is reduced. Under low HBc concentrations, the NTD alone is insufficient to support capsid assembly, and the presence of the CTD is necessary (37, 38). In the absence of CTD, the linker peptide affects the assembly of the NTD and the type of capsid formed. Cp140 predominantly forms T = 3 capsids, whereas Cp149 primarily forms T = 4 capsids. When both NTD and CTD are present, capsid assembly can occur without the linker peptide (18, 39). Heat shock protein 90 (Hsp90) also participates in capsid assembly by binding to Cp149 dimers, which are then packaged into the capsid. The activation of Hsp90 promotes capsid assembly and enhances stability (40).

### 3.2 Functions of CTD in HBV life cycle

**Figure 2:**
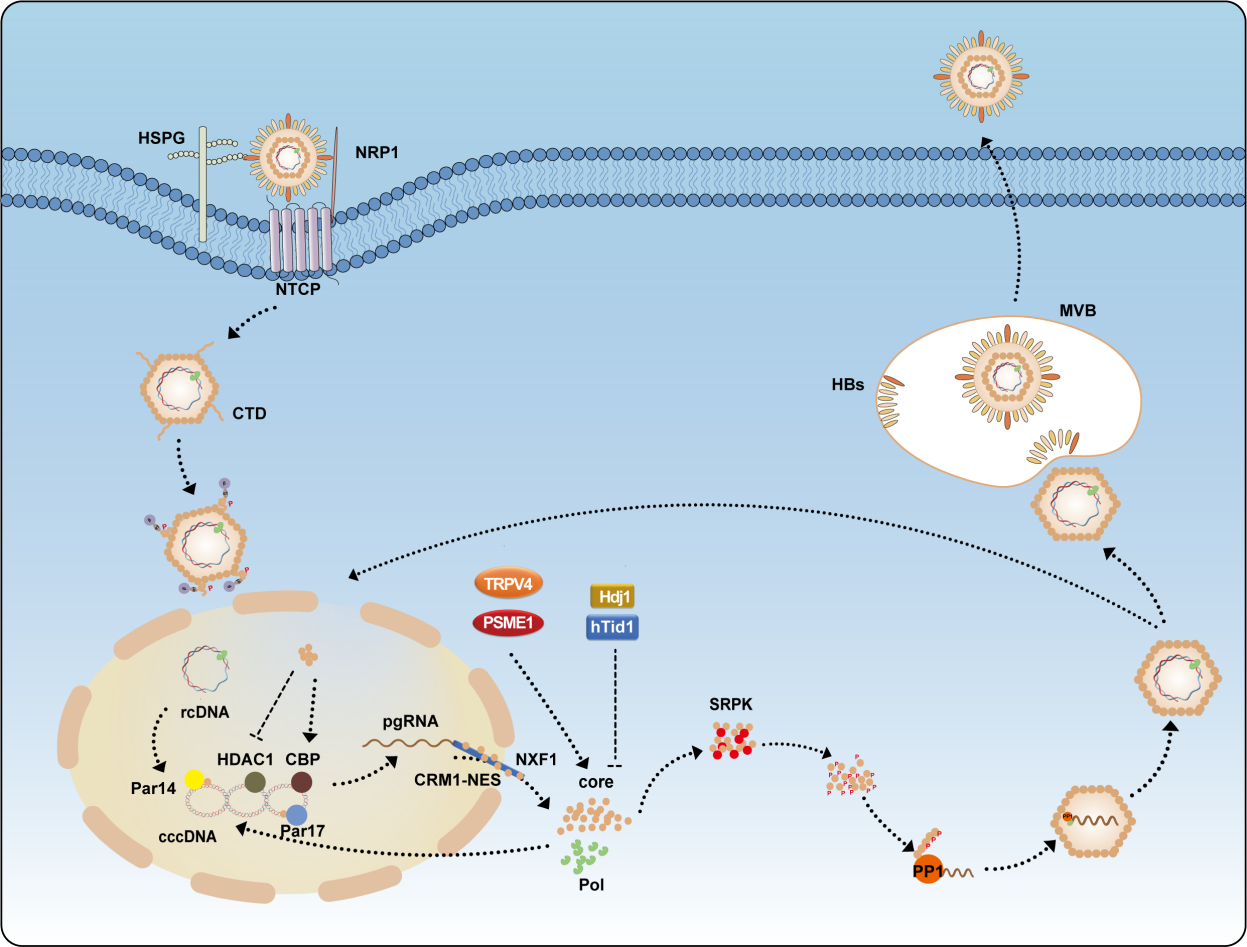
The life cycle of HBV. HBV enters hepatocytes through the receptor NTCP, assisted by HSPG and NRP1. Following entry, CTD is phosphorylated to facilitate its nuclear import. Inside the nucleus, HBc modulates the activity of Par14, Par17, CBP, and HDAC1 on cccDNA to enhance transcription. Newly synthesized HBc, translated from pgRNA, can be exported to the cytoplasm via the CRM1-NES and NXF1 pathways. Its expression is promoted by TRPV4 and PSME1 but inhibited by Hdj1 and hTid1. SRPK binds the unphosphorylated CTD to inhibit capsid assembly; CTD phosphorylation triggers the release of SRPK, allowing PP1 to dephosphorylate the CTD and facilitate nucleocapsid packaging. Mature nucleocapsids are either recycled to the nucleus to replenish the cccDNA pool or enveloped by HBs in MVBs and secreted as infectious virions.CBP, CREB-binding protein; HDAC1, histone deacetylase 1; HSPG, heparan sulfate proteoglycans; HBs, hepatitis B surface antigen; MVBs, multivesicular bodies; NRP1, neuropilin-1; NTCP, sodium taurocholate cotransporting polypeptide; NXF1, nuclear RNA export factor 1; PP1, protein phosphatase 1; PSME1, proteasome activator subunit 1; SRPK, serine-arginine protein kinase; TRPV4, transient receptor potential vanilloid 4.

Although CTD is not essential for capsid assembly, it plays a crucial role in regulating several aspects of the HBV life cycle, including the packaging of pgRNA, genome reverse transcription, and intracellular transport (41). The CTD contains seven serine and one threonine residues that can be phosphorylated, namely Ser155, Ser162, Ser168, Ser170, Ser176, Ser178, Ser181, and Thr160 (42). CTD regulates HBV replication through phosphorylation and dephosphorylation. Serine/arginine protein kinase (SRPK), which binds to the unphosphorylated CTD, inhibits capsid assembly by preventing premature self-assembly. Upon phosphorylation, SRPK’s affinity for HBc decreases, releasing HBc to assemble the capsid, ensuring that HBc assembles at the appropriate time and location. SRPK is removed at the right time to allow capsid self-assembly (43, 44). CTD also interacts with RNA and is essential for the packaging of pgRNA. *In vitro* studies have shown that HBc lacking CTD cannot encapsidate pgRNA, but CTD does not have specificity for pgRNA (37, 41, 45). The Pol protein consists of four structural domains: the terminal protein (TP) domain, spacer domain (SD), reverse transcriptase (RT) domain, and RNase H domain. The tyrosine in the TP domain binds to the ϵ structure of pgRNA to form a P-ϵ ribonucleoprotein (RNP) complex, which recruits HBc protein to assemble the viral capsid. Phosphorylation of CTD facilitates the specific packaging of the RNP complex into the capsid, while preventing non-specific RNA packaging when the complex is absent or limited (16, 46). Studies have shown that CTD is phosphorylated in both dimer and pgRNA-containing capsid forms. Substitution of Ser155, Ser162, and Ser170 with alanine or aspartic acid to mimic dephosphorylated or phosphorylated states affects pgRNA packaging. Phosphorylation of Ser162 is necessary for pgRNA packaging, while phosphorylation of Ser170 optimizes the process (47). Substituting Thr160, Ser168, and Ser176 with alanine to mimic the dephosphorylated state severely impairs pgRNA packaging (42). Interestingly, studies have shown that HBc is phosphorylated in free dimers and empty capsids but is less phosphorylated in nucleocapsids containing pgRNA and DNA, suggesting that dephosphorylation may occur during pgRNA encapsidation (48). Pol recruits protein phosphatase 1 (PP1) (**Figure 2**), which dephosphorylates HBc-Ser170, and PP1 catalytic subunits α and β cooperate with pgRNA and Pol to package the nucleocapsid, promoting pgRNA packaging (49, 50).

Without the HBV capsid, Pol cannot reverse transcribe pgRNA into rcDNA. The core CTD contains 16 arginine residues, with 14 of them grouped into four clusters of three or four. These arginine clusters contribute to the reverse transcription process, including negative strand DNA synthesis extension, primer translocation, cyclization, and positive strand DNA extension (51). The phosphorylation state of the duck hepatitis B virus (DHBV) nucleocapsid dynamically changes during reverse transcription. Immature nucleocapsids (containing pgRNA), mature nucleocapsids (containing dsDNA), and secreted nucleocapsids were isolated from DHBV virus, and it was found that HBc was phosphorylated in immature nucleocapsids, but dephosphorylated in mature and secreted nucleocapsids (52). Phosphorylated HBc promotes negative strand DNA synthesis and the initiation of positive strand DNA extension, and the extension of these strands leads to HBc dephosphorylation. Dephosphorylated HBc, in turn, promotes further maturation of the positive strand DNA and stabilizes the mature nucleocapsid (53). It is hypothesized that the DNA synthesis process is associated with the dephosphorylation of CTD, but studies have shown that dephosphorylation of Ser155, Ser162, and Ser170 decreases the efficiency of negative strand DNA extension, primer translocation, cyclization, and positive strand DNA extension (54). While phosphorylation of Ser155, Ser162, and Ser170 also reduces these steps’ efficiency, the reduction is less pronounced, indicating that phosphorylation is required for rcDNA synthesis (55).

### 3.3 Regulation of cccDNA formation

It has proposed that HBc may negatively regulate cccDNA formation through its involvement in nucleocapsid envelopment and virion secretion by interacting with the viral envelope proteins. This hypothesis is supported by studies showing that mutations in either HBc or the L surface protein, which impair nucleocapsid envelopment and virion secretion, lead to increased cccDNA formation via intracellular amplification (56–61). However, there is no direct evidence to support the notion that HBc itself directly inhibits cccDNA formation. The cleavage of the nucleocapsid is regulated by the dynamic phosphorylation and dephosphorylation of HBc, controlling the release of the rcDNA genome and its conversion into cccDNA. In addition to CTD being phosphorylated, the NTD also has two conserved phosphorylation sites, Ser44 and Ser49. Substituting alanine to mimic the dephosphorylated state did not affect HBc expression, capsid assembly, pgRNA packaging, DNA synthesis, or complete virion secretion, but it inhibited cccDNA synthesis. Substituting glutamic acid to simulate the phosphorylated state partially impaired pgRNA packaging but destabilized the mature nucleocapsid, promoting cccDNA synthesis and enhancing CTD phosphorylation. These results suggest that phosphorylation of the NTD sites should not occur during the early stages of virus assembly but should occur after nucleocapsid maturation to facilitate the next stage of replication, such as during infection or nuclear entry, when nucleocapsid uncoating occurs to promote cccDNA formation (62).

Furthermore, HBc regulates the nuclear import of rcDNA to control cccDNA formation. During nucleocapsid maturation, an increase in negative charges from rcDNA synthesis may induce the exposure of CTD inside the immature nucleocapsid to the surface of the mature nucleocapsid, allowing the nuclear localization signal (NLS) in CTD to function and transport rcDNA into the nucleus for cccDNA formation (63). A study has found that there are two highly conserved lysine residues on HBc, namely K7 and K96, which are not required for HBV replication. However, the codon encoding K7 constitutes part of the RNA polyadenylation signal and is indispensable for cccDNA transcription (64). HBc also participates in cccDNA transcription by binding to cccDNA, reducing the nucleosomal spacing of cccDNA-histone complexes, altering the chromatin structure of cccDNA, and promoting its transcription (65). HBc prefers to bind to regions of cccDNA that are rich in CpG dinucleotide sequences. HBc promotes the binding of the acetyltransferase CREB-binding protein (CBP) to cccDNA and inhibits the binding of histone deacetylase 1 (HDAC1) to cccDNA, promoting histone acetylation and enhancing cccDNA transcription (66). However, studies have also shown that HBc does not participate in cccDNA transcription, neither overexpression nor deletion of HBc affects HBV RNA levels (67, 68).

### 3.4 Immunogenicity and application potential of HBc

HBc has significant immunogenicity during HBV infection, stimulating the immune system to produce specific antibodies. HBc can induce T cell responses, and patients with high frequencies of HBc and Pol-specific T cells can control HBV well after discontinuation of nucleos(t)ide analog (NAs) therapy. Patients who successfully clear the virus typically exhibit strong HBc-specific CTL responses (69, 70). Studies have found that both thymus-dependent and thymus-independent IgM and IgG antibodies can be detected in mice, suggesting that HBc can directly activate B cells to produce antibodies without T cell involvement, although thymus-dependent mice generate more antibodies, facilitating a more effective immune response. This indicates that HBc is both a T cell-dependent and T cell-independent antigen (71). Studies have shown that HBc can specifically bind to the immunoglobulin (mIg) antigen receptor on the surface of resting mouse B cells, and B cells can present HBc to naïve Th cells *in vivo* and to T cell hybridomas *in vitro*, promoting T cell proliferation (72, 73). HBV-specific CD8+ T cells have been shown to play a crucial role in suppressing HBV replication, and C64–72 may be the immunodominant epitope of HBV core antigen, which binds with high affinity to HLA-A*0201 and induces specific CTL responses (74). The protein transduction domain (PTD) of the human immunodeficiency virus type 1 (HIV-1) Tat protein can cross the lipid bilayer of cells, either alone or as a fusion protein. The PTD- HBc fusion protein increases IFN-γ+ CD8+ T cells, enhances CTL responses, and suppresses HBV replication (75).

## 4 Interaction between HBV core protein and host

HBc is a key protein in the viral replication process, and the host can influence viral replication through its interaction with HBc. In hepatocytes, HBc promotes HBV replication by enhancing the binding ability of the nuclear factor κB (NF-κB) dimer p50/p65 to DNA, thereby activating the enhancer II/core promoter (EnII/Cp) (76). Peptidyl-prolyl cis-trans isomerases Par14 and Par17 interact with HBc through the 133RP134 motif, bind to HBc, and are also incorporated into core particles. Par14/Par17 enhance the stability of HBc and core particles and recruit HBc to cccDNA, promoting HBV replication (77). Members of the Hsp40/DnaJ protein family, Hdj1 and hTid1, bind to HBc and promote its degradation, thereby inhibiting capsid formation and HBV replication (78).

The proteasome pathway is the major route for intracellular protein degradation. Transient receptor potential vanilloid 4 (TRPV4) inhibits HBc degradation through a ubiquitin-dependent proteasome pathway, thereby increasing HBc stability, promoting capsid assembly, and enhancing HBV replication. Additionally, TRPV4 can increase H3K4 methylation, further promoting cccDNA-dependent transcription (79). Similarly, proteasome activator subunit 1 (PSME1) interacts with HBc to reduce its binding to the 26S proteasome, inhibiting ubiquitination and enhancing its stability. Moreover, PSME1 increases H3K27 acetylation levels, thereby promoting the transcriptional activity of cccDNA (80) (**Figure 2**).

In summary, HBc not only facilitates intracellular transport of the viral genome and modulates host immune responses to promote viral replication, but it is also recognized by the host. While this recognition can activate antiviral immunity to suppress the virus, it may also lead to excessive immune activation, potentially mediating immunopathological damage to liver tissue.

### 4.1 Intracellular Transport and Localization

During infection, HBc shuttles between the nucleolus and cytoplasm of hepatocytes. The nucleocapsid, with a diameter of 32/36 nm, is close to the size limit for transport through nuclear pores but can still enter the nucleus in its intact form (81). The nucleocapsid is directionally transported to the nuclear pore via the microtubule system (82). Four subcellular localization signals have been identified in the arginine-rich domain (ARD) of HBc. ARD-I and ARD-III synergistically function as nuclear localization signals (NLS), while ARD-II and ARD-IV independently serve as nuclear export signals (NES), both of which are critical for the bidirectional trafficking of HBc or capsids (83–85). The NLS is exposed on the capsid surface through a pore at a quasi-sixfold vertex of the icosahedral structure. importin α1 binds to the NLS, exposing the importin β-binding (IBB) sequence of importin α, which then binds to importin β to form a trimeric complex. Importin β mediates the docking of the complex at the nuclear pore complex (NPC) and facilitates its translocation through the nuclear pore to the nuclear basket. Only intact nuclear capsids can release the genome into the nucleus (86–90).

Phosphorylation of the CTD (C-terminal domain) is an essential step for transporting the viral genome into the nucleus. Studies have shown that phosphorylation at Ser155, Ser162, and Ser170 promotes CTD compaction, facilitates the externalization of CTD, and exposes the NLS on the capsid without affecting its affinity for importin α1 (89, 91).

The nuclear export of HBc and capsids depends on two different pathways. The ARD of HBc interacts with NXF1 (nuclear export factor 1), mediating the nuclear export of mRNA and proteins (85). Each tip region of the HBc particle contains two CRM1 (chromosome region maintenance protein 1)-dependent nuclear export signals (NES^CRM1), and there are 480 NES^CRM1 signals on the surface of a capsid. This high density of NES^CRM1 signals enables efficient binding of HBc particles to CRM1, facilitating nuclear export of the capsids. Additionally, CRM1 facilitates the secretion of mature nucleocapsids from the nuclear pores to the endoplasmic reticulum/Golgi apparatus for viral particle formation by coupling with the microtubule system (92, 93).

HBc monomers depend on the NXF1 pathway to be transported from the nucleus to the cytoplasm, participating in pgRNA packaging. Nucleocapsids rely on the CRM1 pathway to be exported from the nucleus to the cytoplasm for reverse transcription and viral assembly.

### 4.2 Regulation of host immune responses

The innate immune response serves as the first line of defense against invading pathogens. Pattern recognition receptors (PRRs) detect pathogens and induce the production of antiviral factors to control HBV infection. Moreover, the innate immune response is crucial for activating the adaptive immune response, as it mediates the recruitment of adaptive immune cells. T cells and B cells recognize and eliminate HBV-infected hepatocytes. However, persistent exposure to viral antigens can lead to immune exhaustion, which eventually establishes chronic infection by dampening host responses (94–96).

**Figure 3:**
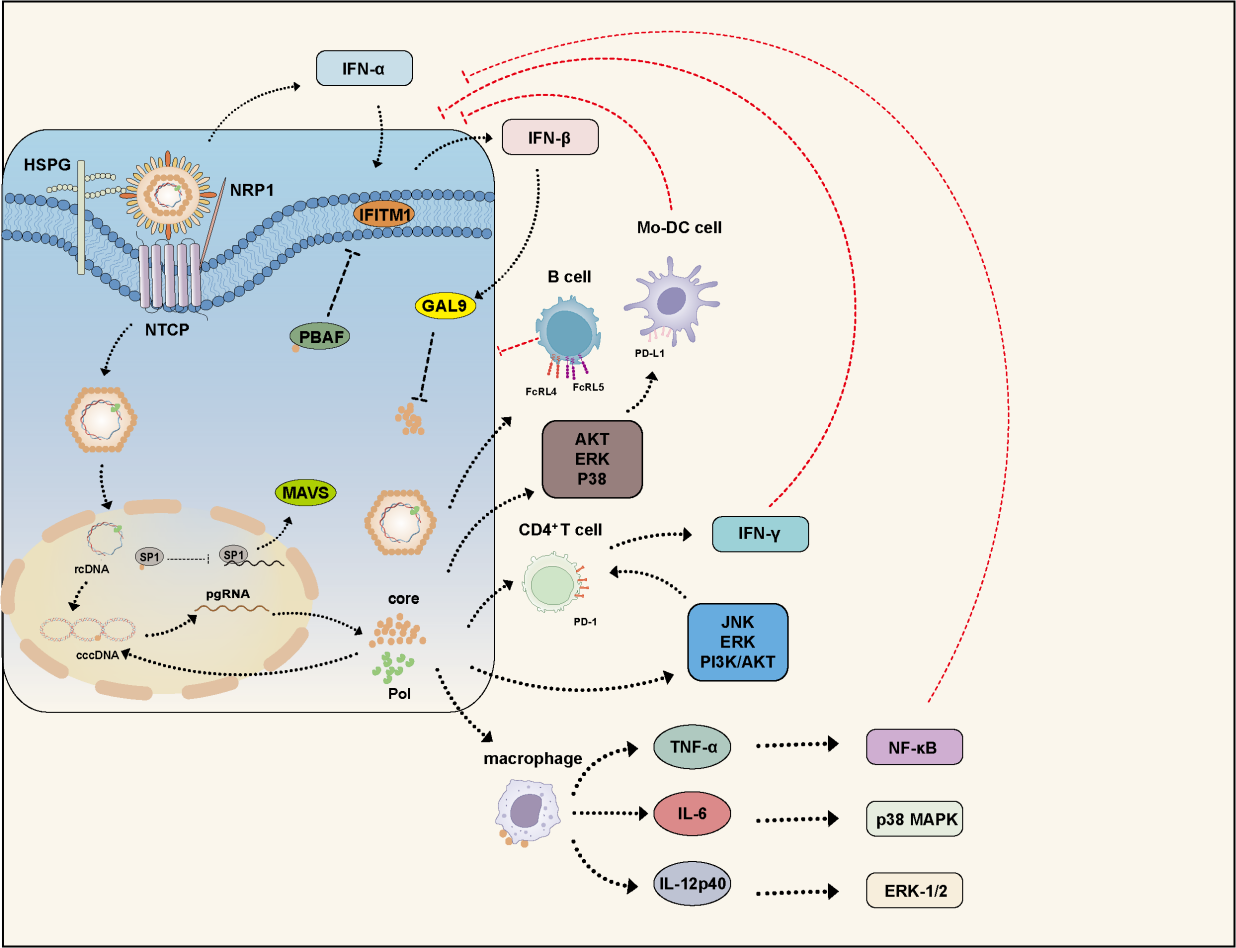
HBc modulates the host immune response. HBV infection activates multiple IFN signaling pathways. IFN-α induces the expression of IFITM1, while IFN-β upregulates GAL9 to inhibit HBV replication. However, HBc counteracts these defenses by disrupting the stability of PBAF, thereby suppressing IFITM1 expression. Additionally, HBc interacts with the transcription factor SP1 to suppress MAVS expression, leading to inhibition of the IFN-β signaling pathway. Furthermore, HBc participates in various signaling pathways and impairs the functions of B cells, Mo-DC, CD4+ T cells, and macrophages, thereby facilitating HBV persistence. GAL9, Galectin-9; PBAF, polybromo-associated BAF; IFITM, interferon-induced transmembrane protein; MAV, mitochondrial antiviral-signaling protein.

The capsid, via its arginine-rich domain, binds to heparan sulfate on macrophage surfaces and induces the production of cytokines such as TNF-α, IL-6, and IL-12p40. This process involves activation of NF-κB, ERK-1/2, and p38 MAPK signaling pathways, thereby enhancing immune responses and activating adaptive immunity (97). HBV infection can trigger the production of interferons (IFNs) and induce interferon-stimulated genes (ISGs) to exert antiviral effects (98). IFN-β can induce the expression of galectin-9 (GAL9), which, mediated by another ISG-encoded protein, viperin, interacts with HBc and promotes HBc accumulation in the cytoplasm. Interestingly, the autophagosome marker LC3 colocalizes with HBc. Further studies revealed that GAL9 enhances the auto-ubiquitination of E3 ubiquitin ligase RNF13, promoting p62 recruitment and interaction with LC3 to form autophagosomes, leading to selective autophagy of HBc (99) (**Figure 3**).

HBc also suppresses MAVS (mitochondrial antiviral-signaling protein) expression by interacting with the transcription factor SP1 and inhibiting its binding to the MAVS promoter. MAVS activation can suppress HBV replication via IFN-β induction, and combined use of MAVS and IFN-α has been shown to enhance antiviral effects both *in vitro* and *in vivo*. Our group has identified several ISGs that inhibit HBV by targeting the HBx protein, However, IFN-α treatment alone is not fully effective against HBV infection, as IFN-α also induces the RNA-editing enzyme ADAR1, which targets the 3’UTR of MAVS mRNA to suppress its expression (100–108). Interestingly, research found that IFN-induced IFIT3 significantly promotes HBV replication, suggesting that IFN may also activate certain ISGs that enhance viral replication (109). Early studies found that during acute HBV infection in chimpanzees, there was no induction of genes associated with innate immune responses. HBV remains undetected and spreads before the adaptive immune response initiates, effectively evading recognition by the innate immune system during early infection (110, 111).

The BRG1-associated factor (BAF) and polybromo-associated BAF (PBAF) are mammalian SWI/SNF chromatin remodeling complexes. They regulate the expression of various interferon-inducible genes via ATP-dependent chromatin remodeling. BAF200 is a specific subunit of PBAF. HBc disrupts PBAF stability by interacting with BAF200, thereby suppressing the expression of interferon-induced transmembrane proteins (IFITMs) and partially restoring HBV replication despite IFN-α-induced immune responses (112) (**Figure 3**).

Phosphorylation of serine residues within the arginine-rich domain of HBc inhibits the binding of capsids to macrophages and the secretion of cytokines. HBc remains dephosphorylated during asymptomatic infection but is phosphorylated during viral replication in hepatocytes, indicating that HBc can escape immune responses via phosphorylation modifications (97, 113). In HBV-infected patients (especially those with acute hepatitis), HBc can promote the proliferation of CD4+ T cells and stimulate the production of IFN-γ and lymphotoxin, thereby regulating immune responses and facilitating HBV clearance (114). Deletion of HBc leads to high levels of HBsAg in mice, with 93.3% of mice remaining HBsAg positive after 12 weeks. A C-terminal deletion of 10 amino acids in HBc abolishes effective HBc-specific IFN-γ responses, promoting HBV persistence (115).

Programmed death receptor 1 (PD-1) overexpression is a hallmark of exhausted T cells. HBc induces PD-1 expression on CD4+ T cells via JNK, ERK, and PI3K/AKT signaling pathways. PD-1 expression correlates with HBV DNA levels during the immune clearance phase, influencing viral clearance and contributing to HBV persistence (116). Programmed death ligand 1 (PD-L1, also known as B7-H1) binds to PD-1, transmitting co-inhibitory signals to T cells and regulating their activation and tolerance. HBc upregulates B7-H1 expression in monocyte-derived dendritic cells (Mo-DCs) through activation of AKT, ERK, and p38 pathways, leading to Mo-DC apoptosis and impaired HBV DNA clearance (117). Fc receptor-like proteins 4 (FcRL4) and 5 (FcRL5) are inhibitory receptors on B cells that regulate immune responses by suppressing B cell activation and proliferation. HBc binds specifically to B cells, inducing high expression of FcRL4 and FcRL5, thereby impairing B cell function (118) (**Figure 3**).

Overall, HBc modulates both innate and adaptive immune responses, enabling immune evasion and contributing to viral persistence.

### 4.3 HBc and host cell cytokines

The immune system acts as a double-edged sword: while it clears infected cells, it also triggers inflammation and necrosis in liver tissue, leading to the destruction of infected hepatocytes. Prolonged chronic inflammation can result in liver fibrosis, cirrhosis, and even hepatocellular carcinoma. Many cytokines are associated with adverse outcomes in hepatitis B. HBc, which is highly immunogenic, can induce the production of various cytokines, contributing to disease progression in hepatitis B.

HBc promotes the expression of NLRP3 (NOD-like receptor family pyrin domain-containing 3), a component of the NLRP3 inflammasome, and activates caspase-1, leading to the secretion of IL-1β and IL-18 through the NLRP3 inflammasome pathway, thereby triggering liver inflammation (119). HBc also enhances the expression and secretion of IL-6 by activating the ERK, p38 MAPK, and NF-κB signaling pathways (120).

In the presence of IL-6, transforming growth factor-β (TGF-β) promotes the differentiation of naïve T lymphocytes into Th17 cells, thereby fostering autoimmunity and inflammation (121–124). Cytokines secreted by Th17 cells, particularly IL-17, are associated with the progression of liver fibrosis and cirrhosis. In patients with hepatitis B, IL-17 is mainly found in fibrotic areas, and its levels closely correlate with the severity of fibrosis (125).

IL-17 activates hepatic stellate cells (HSCs) by interacting with IL-17 receptors (IL-17R) on their surface. Additionally, IL-17 can activate HSCs via the p38 and ERK1/2 signaling pathways. It also stabilizes TGF-β receptor II in a JNK-dependent manner, thereby activating the Smad2/3 pathway to further stimulate HSC activation and enhance liver fibrosis (126, 127).

## 5 HBc clinical relevance

### 5.1 Clinical applications of anti-HBc

Anti-HBc is a classical serological marker. Once infected with HBV, Anti-HBc usually persists for a long time, often for life. Therefore, Anti-HBc is the most useful marker for determining past exposure to or infection with HBV (128). Anti-HBc IgM is an early marker of HBV infection, typically appearing 6 to 8 weeks after infection and peaking during the acute phase. However, it gradually disappears within 6 months after infection. Anti-HBc IgM levels can also rise during acute exacerbations of chronic hepatitis, though the titers are lower compared to those during acute hepatitis, allowing differentiation between acute and chronic infections (129).

Anti-HBc IgG follows Anti-HBc IgM, usually appearing 4 to 8 months after acute infection and persisting for a long time. A positive Anti-HBc IgG result indicates past HBV infection, which could either be a resolved past infection or a chronic infection (130, 131). If both HBsAg and Anti-HBs are negative while Anti-HBc is positive, several scenarios must be considered, such as false positivity, the window period of acute infection, a recovered past infection with declining antibody levels, occult infection, co-infection with other hepatitis viruses, or mutations in HBsAg (132, 133). Therefore, in clinical diagnostics, Anti-HBc testing is typically combined with other markers (such as HBsAg, HBeAg, and HBV DNA) to improve diagnostic accuracy and predictive power. Anti-HBc IgG is a surrogate indicator of HBV-specific activation of adaptive immune response (134). Anti-HBc IgG includes four subtypes: IgG1, IgG2, IgG3, and IgG4. Studies have reported that in chronic carriers, IgG1 > IgG3, whereas in recovered individuals, IgG3 > IgG1 (135, 136). HBeAg and HBc are translated from different start codons within the C ORF and share an identical 149-amino-acid core domain (137). HBeAg is a serum marker, which is closely related to the number of infectious hepatitis B virus particles in the serum (138).HBeAg suppresses T-helper (Th) cell function, blocks the IgM-to-IgG class transition, and significantly reduces the production of anti-HBc IgG. It also induces T-cell tolerance to diminish the immune response against intracellular HBc, thereby promoting persistent viral infection (139).

The hepatitis B vaccine, based on recombinant DNA technology to express HBsAg, protects against HBV infection by stimulating humoral immune responses; thus, vaccinated individuals develop only anti-HBs antibodies (140). For patients who are solely Anti-HBc positive, vaccination can still elicit a good immune response. Moreover, their response to vaccination may provide additional diagnostic information: patients who respond to the vaccine may have had a false-positive Anti-HBc, while non-responders may harbor occult HBV infection (133, 141).

Quantitative Anti-HBc (qAnti-HBc) levels vary significantly across different stages of HBV infection and are determined by the host’s immune status. qAnti-HBc can more accurately reflect hepatic inflammation and is associated with the degree of liver fibrosis, exacerbation during chronic infection, and the presence of occult infection. The qAnti-HBc level can serve as a predictive marker for spontaneous or treatment-induced HBeAg and HBsAg seroclearance, relapse after treatment cessation, reinfection post-liver transplantation, and viral reactivation during immunosuppression (142, 143).

Anti-HBc and qAnti-HBc each have distinct clinical focuses: Anti-HBc is mainly used for preliminary screening and long-term monitoring of HBV infection, whereas qAnti-HBc offers significant advantages in assessing hepatic inflammation, predicting treatment responses, and estimating relapse risk.

### 5.2 HBc variants and drug resistance

Nucleoside analogs (NAs) inhibit viral replication by suppressing the function of the Pol, but the rate of sustained virological response is very low, requiring long-term treatment. Long-term NA therapy increases the risk of antiviral drug resistance. Resistance mutations primarily occur in the Pol, but studies have shown that HBc mutations can compensate for the replication defects of drug-resistant HBV, enhancing the virus’s survival ability under drug pressure.

HBc mutations (P5T, S35T, P79Q, E83D, S87G, I97L, and Q177K) were found in patients with lamivudine (LMV) resistance, promoting the packaging of pgRNA and thereby enhancing HBV DNA replication, with the P5T mutation playing a significant role (144). In patients undergoing NA therapy, an increased frequency of the P130L mutation has been observed. Researchers speculate that the P130L mutation may alter the secondary structure of HBc, reducing its antigenicity and preventing immune recognition, leading to immune escape. However, this hypothesis requires further experimental validation (145) (**Table 1**).

In summary, nucleotide analog resistance mutations do not occur solely in the Pol; compensatory mutations in HBc can also promote HBV replication.

### 5.3 HBc as a therapeutic target

**Figure 4:**
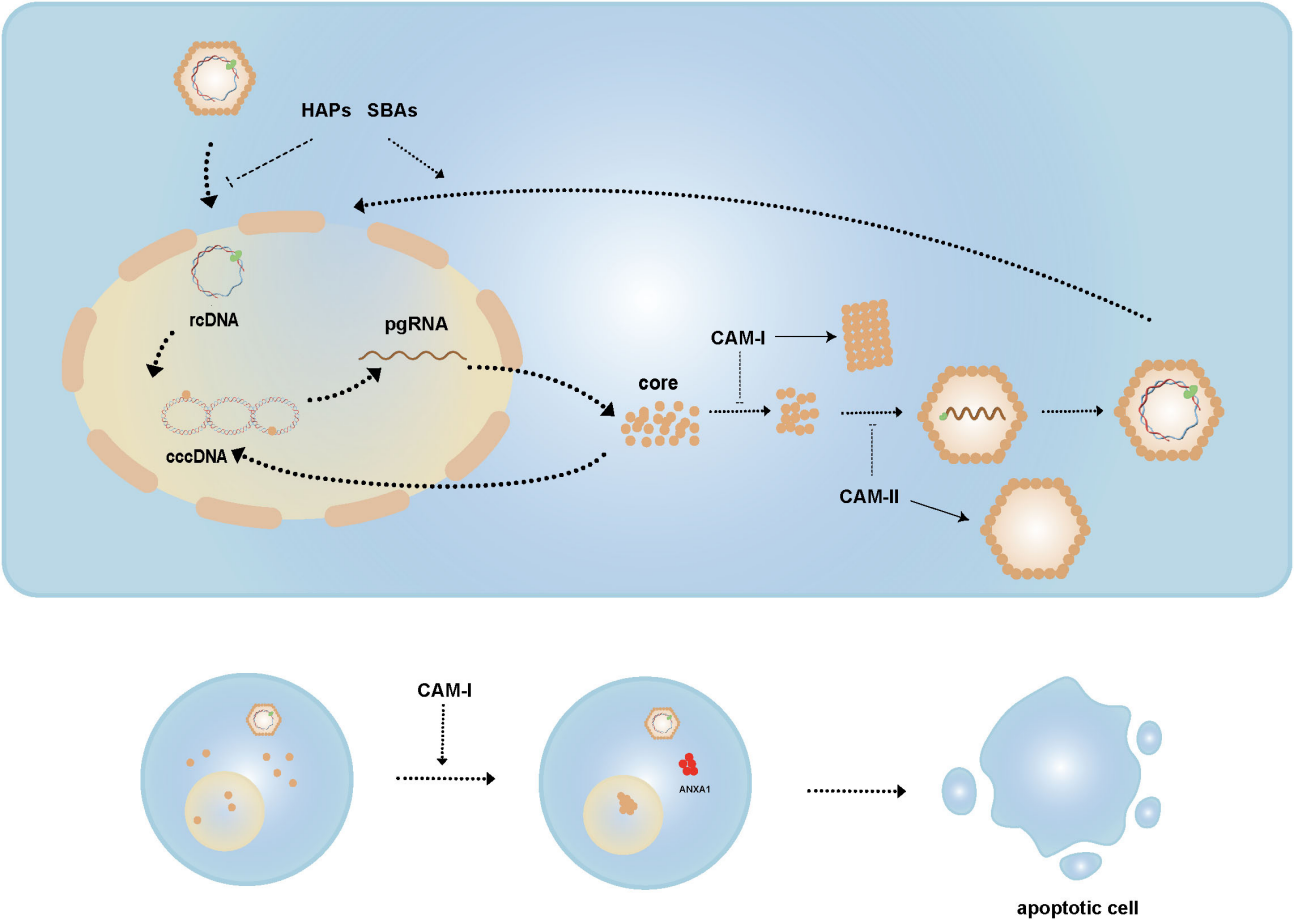
Mechanisms of CAMs. CAM-I acts by altering capsid conformation, whereas CAM-II interferes with pgRNA packaging to generate empty capsids. HAPs and SBAs affect nucleocapsid disassembly, inhibiting this process during infection but inducing it in mature nucleocapsids. CAM-I induces nuclear aggregation of HBc and promotes ANXA1 expression, ultimately leading to apoptosis of HBV-infected cells.

HBc plays an indispensable role in multiple steps of the HBV life cycle. Therefore, several drugs aim to target HBc to disrupt various stages of the replication cycle. Capsid assembly modulators (CAMs) are antiviral agents that interfere with the dynamics and morphology of the capsid (**Figure 4**). Based on their functions, CAMs are classified into two types:

- CAM-A, such as heteroaryl pyrimidines (HAPs), induce the misassembly of HBc dimers, leading to aberrant capsid structures and HBc aggregation.
- CAM-E, such as sulfonylbenzamide (SBAs), phenylpropenamide (PPA), pyrazolyl thiazole (PT), ethoxamide-pyrrolamide (GPA), and dibenzothiazepine-2-one (DPT), stabilize dimer-dimer interactions to form intact capsids that lack the viral genome (146, 147).

CAMs disrupt HBc assembly by binding to a hydrophobic pocket at the dimer interface, enhancing dimer-dimer interactions. CAM-A induce HBc phosphorylation, altering the capsid’s conformation and affecting its stability, whereas CAM-E interfere with the packaging of pgRNA, resulting in the formation of empty capsids (35, 148).

CAMs not only affect capsid assembly but also play dual roles during HBV replication. Studies have shown that HAPs and SBAs not only impair capsid assembly but also induce the disassembly of viral particles and cytoplasmic mature nucleocapsids, thereby regulating the formation of cccDNA. Specifically, by triggering the disassembly of nucleocapsids in viral particles, CAMs prevent rcDNA from entering the nucleus and inhibit cccDNA synthesis during new infections. Conversely, by inducing the disassembly of mature cytoplasmic nucleocapsids, they accelerate rcDNA nuclear import and conversion to cccDNA (149).

Additionally, research has found that high concentrations of HAP12, in collaboration with importin β, tend to promote the disassembly of HBV empty capsids (150). Some studies suggest that CAMs induce the aggregation of HBc within the nucleus, promoting the expression of apoptosis-related gene ANXA1 and activating the interferon signaling pathway, thereby helping to clear HBV-infected cells. However, CAM-induced cell death was not observed in HBV-infected primary human hepatocytes (PHHs), likely because HBc aggregates form a complex with the STIP1 homology and U box-containing protein 1 (STUB1) and heat shock protein 70 (Hsp70), recruit Bcl-2 associated athanogene 3 (BAG3) to transport them to the perinuclear region, and are subsequently degraded via the ubiquitin-binding protein p62-mediated autophagosome formation (147, 151–153).

Due to the inability of existing drugs to eliminate cccDNA and the emergence of drug resistance, CAMs, by intervening at multiple steps of the viral life cycle, may offer a more durable antiviral effect and reduce the risk of resistance, making them a focus of current drug development efforts. In recent years, several CAMs have entered clinical development stages.

RO7049389, a HAP compound, induces aberrant capsid assembly leading to degradation of the viral core protein, thereby inhibiting pgRNA packaging and HBV DNA replication (154). It is currently in Phase 2 clinical trials. Results from Phase 1 trials showed that after 4 weeks of treatment in patients with chronic hepatitis B (CHB), RO7049389 led to a reduction in HBV DNA with infrequent and mild adverse events, comparable to those in the NAs control group (155). Additionally, given the high prevalence of HBV in Asia, the safety of RO7049389 was validated in healthy Chinese volunteers during Phase 1 trials (156).

Studies have shown that ABI-H2158 promotes abnormal capsid assembly, blocks pgRNA encapsidation, inhibits HBV DNA replication, and prevents cccDNA formation by inducing premature disassembly of capsids during infection. Although ABI-H2158 had entered Phase 2 clinical trials, development was halted due to hepatotoxicity (157).

JNJ-6379, similar in mechanism to ABI-H2158, is a CAM-E evaluated for safety, pharmacokinetics, and antiviral activity in Phase 1 clinical studies. Data indicated that JNJ-6379 was well tolerated with mostly mild to moderate adverse events. HBV DNA and RNA levels decreased in a dose-dependent manner, reaching levels below the lower limit of quantification at the highest doses. However, there was no significant effect on HBsAg and HBeAg levels, and viral rebound was observed after treatment cessation (158).

Phase 2 studies found that virologic breakthrough could occur when JNJ-6379 was used as monotherapy, but this was not observed when combined with NAs. The combination significantly enhanced the reduction of HBV DNA and HBV RNA levels, though the impact on HBsAg and HBeAg remained limited (159).

JNJ-3989, a small interfering RNA (siRNA), reduces levels of all HBV proteins and pregenomic RNA by targeting and cleaving HBV RNA transcripts. In Phase 2 studies, the combination of JNJ-6379, JNJ-3989, and NAs not only maintained their respective functions but also significantly reduced HBsAg levels and minimized HBV DNA rebound after treatment cessation (160).

## 6 Future research directions and challenges

The ultimate goal in treating hepatitis B virus (HBV) infection is the complete eradication of the virus, ideally through the elimination of covalently closed circular DNA (cccDNA). However, current therapies struggle to effectively clear cccDNA. At present, the most achievable treatment goal is considered to be a functional cure, characterized by the sustained absence of detectable HBsAg and HBV DNA in serum (with or without seroconversion of HBsAg), improvement in liver inflammation and fibrosis, and long-term maintenance of these outcomes after cessation of therapy.

Clinical studies have demonstrated that CAMs can significantly reduce HBV DNA levels, but they exert limited effects on the established cccDNA pool. CAMs function by binding to the hydrophobic pocket at the HBc dimer-dimer interface; thus, mutations within HBc could potentially impact CAM efficacy. JNJ-6379 has shown good antiviral activity against clinical isolates from HBV genotypes A–H, with the exception of one genotype D strain, which exhibited substitution at position 33 of HBc—a site located within the CAM binding pocket (161).

Residue T109, located within the CAM binding pocket, is one of the most frequently mutated sites in HBc. Mutations T109I and T109M promote normal capsid assembly, rendering them relatively resistant to CAMs, while the T109S mutation impairs capsid assembly and increases sensitivity to CAMs (162, 163). Mutations at residues D29G, T33N/S, and Y118F within the CAM binding pocket significantly reduce the antiviral activity of BAY41-4109, whereas mutations at S106T, T128I, and L140I enhance its antiviral efficacy (**Table 1**). These mutations alter the characteristics of the binding pocket, thereby affecting drug binding and antiviral potency. Similarly, other studies have confirmed that numerous amino acid mutations within the CAM binding pocket impact CAM activity (161, 164).

The hydrophobic nature of the amino acids within the CAM binding pocket is crucial for maintaining drug functionality. Interestingly, research has identified a second binding pocket on the HBV capsid, located at the spike apex. This pocket, located at the tip of the capsid spikes and targeted by the peptide dimers, can induce HBc aggregation, thereby interfering with capsid formation and viral assembly (165). This secondary pocket also represents a potential target for future drug development.

## 7 Conclusion

HBc is an indispensable and multifunctional protein within the HBV life cycle, playing critical roles in viral replication, nucleocapsid formation, and persistent infection through complex interactions with host cellular pathways. These interactions not only facilitate viral survival but also contribute to immune evasion and hepatocarcinogenesis, positioning HBc as a pivotal therapeutic target.

Despite these advancements, several research challenges remain. One major obstacle is the limited impact of current CAMs on pre-existing intrahepatic cccDNA pools, which are central to HBV persistence. Additionally, HBc mutations within the CAM-binding hydrophobic pocket can significantly alter drug susceptibility, leading to variable therapeutic responses and potential resistance. The heterogeneity of HBV genotypes further complicates drug development, as genotype-specific mutations (e.g., at positions T109 and T33) may influence HBc structure and CAM efficacy.

Future research should focus on three main directions: (1) the development of next-generation CAMs with broader genotype coverage and improved potency against mutant HBc variants; (2) deeper mechanistic insights into HBc’s role in cccDNA maintenance and host epigenetic modulation, aiming to discover novel therapeutic leverage points; and (3) the integration of CAMs into rational combination regimens that include immune-based therapies, with the goal of achieving a complete cure, defined as the elimination or permanent silencing of cccDNA and integrated HBV DNA.

Ultimately, while functional cure remains a realistic near-term objective, the complete eradication of HBV will require innovative strategies to overcome the resilience of cccDNA, understand host-virus dynamics at a deeper level, and preempt or overcome resistance pathways. HBc-targeted research remains central to this endeavor.
